# Dublin Medical Transactions, Vol. I.—New Series

**Published:** 1831-04-01

**Authors:** 


**Dublin Medical Transactions; a Series of Papers by Members of the Association of Fellows and Licentiates of the King and Queen's College of Physicians in Ireland. New Series, Vol. I. Part I. 1830.**

If the metropolis of the Sister Kingdom has evinced a remarkable anomaly, as respects metropolitan cities in general, in being without a periodical journal of medicine, it has, in a great measure, made up for this defect, of late years, by the issue of several very valuable volumes of Transactions. These, indeed, may be considered as annual medical journals dedicated solely to original communications, and unconnected with the other departments of periodical literature. We agree with the editors of the present series, that by such a publication, "many useful essays have been rescued from oblivion, and many able practitioners have been invited to observe and to inquire." They have to regret the decease of one of their most able and zealous coadjutors and fathers of the work, Dr. Brooke, whose funeral eulogy was pronounced—or at least composed by Dr. Morgan.

Though the present volume be not quite equal to some of its predecessors, it is highly respectable, both as to the names of the contributors and the materials of the communications. In this first notice we shall select a batch of short papers for analysis, throwing the longer and more important ones into separate articles.

### I. *Two Cases of Recovery from Laceration of the Uterus and Vagina.* By Robert Collins, M.D.

*Case* 1. A female, aged 25 years, was admitted into the Lying-in Hospital of Dublin, with the hand and arm of the child protruding out of the vagina, as far as the elbow. It was reported that the midwife had mistaken the hand for the foot, and pulled it down. When admitted, she was in a very debilitated state, with feeble pulse, ghastly countenance, and great anxiety. Rupture of the uterus or vagina was suspected. She had got 60 drops of laudanum before she was admitted.

"On examination, per vaginam, the shoulder and body of the child were found to be forced so low, and so firmly fixed in the pelvis, as to completely forbid any attempt to turn the child, nor was there any necessity to do so, as it was dead.

The thorax of the child was then perforated and broken down, and the breech was afterwards brought down with the crotchet, without the least difficulty.

On introducing the hand into the vagina after the child and placenta were taken away, an extensive laceration was found at the junction of the cervix uteri with the vagina posteriorly.

I placed the parts as nearly as possible in their natural position, guarding carefully against any portion of the intestines being included in the lacerated part, at the same time removing as much of the clotted blood as could be got away.

It was evening when she was admitted into the hospital, and after the delivery, &c. was completed, and her bed made dry, she was ordered a powder containing ten grains of calomel, and the same quantity of jalap, which remained on the stomach, and operated freely on the bowels." 2.

Next morning there was much tenderness of abdomen, and 36 leeches were applied, succeeded by the warm-bath, fomentations, &c. by which much relief was obtained. The pulse gradually returned to its natural state, and she left the hospital perfectly well, on the 23d day after delivery. After three months she presented herself in perfect health. There were no other means used in this case, except attention to the bowels and diet.

*Case* 2. Ann Wood, aged 30, was admitted in labour of her sixth child, at six o'clock in the evening of the 27th October, the labour having commenced seven hours previously.

" When she came into the hospital the uterus was acting briskly, and the head of the child advanced rapidly, so much so, that those who were in attendance thought it would have been expelled every pain. Suddenly, however, the uterine action completely ceased, and considerable debility, great distress of countenance, vomiting, and other symptoms strongly indicating rupture of the uterus ensued.

The head of the child was low down, and pressing on the neck of the bladder, so much so, that the catheter could not be introduced, and as immediate delivery was necessary, the head was lessened, and the child brought away with the crotchet. The uterus assisted strongly in expelling the child and placenta; however, on introducing the hand into the vagina afterwards, a most extensive laceration was found at the junction of the cervix uteri with the vagina anteriorly, and the intestines had fallen through the opening into the vagina.

After returning the intestines carefully, the edges of the laceration were brought as nearly as possible in contact, and the patient was enjoined to remain perfectly quiet during two hours on the couch where she was delivered. She was then cautiously carried to bed, and a powder containing eight grains of calomel and fifteen of jalap, with half a grain of powdered opium, administered." 5.

29th October. The pulse was 114 and feeble—had rested badly—abdomen distended, and tender on pressure—bowels not opened. Castor oil and tincture of jalap were prescribed internally, and 36 leeches were applied to the abdomen, after which she was put into the warm-bath. In the course of the same day the leeches were repeated, and various other means were used to keep down inflammation. We need not follow up the dismal details of the treatment, which was apparently energetic and judicious. It is sufficient to say that, on the 15th and 16th days after delivery, there was a very considerable discharge of unhealthy pus from the vagina, to the amount of a pint in the first instance, but less in the second. This, however, did not interfere with the recovery, and, after the first twelve days, she took nourishment liberally, with tonics and acids.

" In the majority of cases where laceration of the vagina or uterus takes place, the bowels yield with difficulty to the effects of medicine, and in many instances it will be found quite impracticable to purge the patient, with the largest doses of the most drastic purgatives, until death is near at hand, and then the medicines begin to act violently.

It is an object of the greatest importance, in such cases, to have the bowels early opened, and afterwards to keep up their action by mild purgatives, at the same time using every means in our power to counteract inflammation, and no means are more likely to do so, than those already mentioned.

In both cases, it may be observed, that the same plan of treatment was pursued; and it cannot be too strongly recommended to the notice of professional men, that early and active means of counteracting the dangerous and sudden inflammation, that sets in, in all cases of this kind, is a matter of the utmost importance.

In the above instances, when the tenderness of the abdomen was subdued, the dangerous symptoms gradually subsided, and it is singular, that in both it was nearly removed about the end of the fourth, or in the course of the fifth day after delivery.

If possible, the practitioner should avoid letting the child escape out of the uterus, into the cavity of the abdomen in the delivery; sometimes it unavoidably does escape as soon as the accident takes place, but in many instances it may be prevented, by using caution during the delivery, particularly in those cases where we perforate the head.

The opening should be made as much at the side as we can, so as to cause the opposite side of the head to press against the pelvis, and at the same time having an assistant to press strongly on the abdomen of the patient, to keep the uterus as fixed as possible. The perforator should not be pressed with great force against the head, lest it should suddenly recede.

In cases of this kind, attempts have been made to deliver with the forceps, but the introduction of the blades generally forces the child's head out of reach. The child dies also shortly after the laceration takes place, and the dimensions of the pelvis are often defective—all which circumstances prove their inutility in most cases.

When the child escapes out of the uterus into the cavity of the abdomen, it is now the general practice, and undoubtedly the best, to introduce the hand cautiously through the lacerated parts into the abdominal cavity, and bring down the feet of the child, and the sooner this is done after the accident the better. In such cases, much care should be taken to return any of the intestines that fall through the lacerated part into the vagina, otherwise strangulation may take place." 9.

Some cases occur, where the laceration takes place previous to dilatation of the os uteri. Here, it is thought, the patient would have a better chance of recovery by opening the parietes of the abdomen, cutting into the uterus, and extracting the fœtus. This is so rare a case, that no instance of it has occurred since Dr. Collins has been attached to the hospital; nor is there any instance of it recorded by Dr. Clarke, in his valuable abstract, during his seven years' mastership. Generally, the os uteri is so much dilated by labour pains, as to enable the attendant to introduce his hand without difficulty.

A third case of recovery from laceration of the vagina, under similar treatment, occurred lately in a poor woman out of the hospital, and where the laceration was so extensive, that the hand passed into the cavity of the abdomen without the least interruption. The patient lived till the 29th day from the injury, when she was suddenly attacked with hæmorrhage, and died in 15 minutes. She was walking about for four or five days previous to death—had a good appetite—and was, in other respects, tolerably well in health. No examination could be obtained. Many cases of vaginal and uterine laceration have proved fatal during the last three years; but, as our author is preparing an abstract of all the different cases that have occurred, with some practical observations, he abstains from noticing them at present. Eight thousand women were delivered at the above institution during the period alluded to.

II. *Two Cases of Pulmonary Apoplexy, illustrative of the Value of Mediate Auscultation.* By JOHN C. FERGUSON, A.M. M.B. &c.

The first case was that of John M'Cleary, a sailor, aged 36 years—a robust man. Through the past Winter and Spring, Mr. F. had given this man some medicines of an aperient and expectorant quality—the latter for bronchitis. He applied at the Coles' Lane Dispensary on the 19th of June, 1829, complaining of costiveness and cough, with oppresion about the chest, sanguineous expectoration—pallor of countenance—cold, clammy perspiration—feeble pulse at 90. Purgatives were prescribed.

" Trusting to what I knew of his history, and to former examinations of his chest, I made no Stethoscopic examination this day, (a circumstance which I shall ever regret,) and we agreed in attributing the exacerbation of his general symptoms, as perhaps we were justified in doing, to some previous excess. I heard no more of him until sent for in haste on the Sunday morning following. I found him dead; and it is worthy of remark, that although two hours had not elapsed since his decease, the rigidity of the body was considerable. A neighbouring apothecary had attempted to bleed him, but unsuccessfully. I was told he had passed the preceding night rather restlessly; however, he had his breakfast of soup and bread, and in the act of putting on his shoes, complained to his wife of loss of vision; seemed to faint, and died without a struggle. During Saturday, he had expressed himself relieved by the operation of the purgative medicines, and, what is a remarkable fact in the history of this case, he had expectorated no blood for fourteen hours before death, nor in the agony was there any escape of blood from the mouth or nares, which might lead to a suspicion of the real seat of disorganization.

Examination forty-eight hours after death, in which I have to acknowledge the valuable assistance of my friend Dr. Law.

The body of a robust man; putrefaction had considerably advanced, particularly about the neck and shoulders, where a degree of emphysema was produced by it.

HEAD.—Not examined.

CHEST.—The left pleural sack contained about three quarts of blood; the serum supernatant to a great degree, as in blood allowed to stand after venesection, and the clot in considerable quantity, but very soft, occupying the most dependent portion of the cavity. This lung had contracted no adhesions, and on carefully raising it out of its situation, and dissecting it out by its root, the following appearances presented themselves to us. The superior lobe was one mass of the most perfect pulmonary apoplexy; the structure of the lung seeming to be absolutely broken up by the excessive effusion of blood into it. The appearance which this disorganized part put on differed, however, in some respects, from the description of pulmonary apoplexy given by Laennec. Speaking of this form of disease, he says,—' This alteration of structure consists in a degree of hardening of the lung, equal to that of its most extreme state of hepatization; but in other respects quite different. It is always partial, and but seldom engages a large portion of the lung.' Now, in the present case, so far from resembling the firmness of an hepatized lung, the apoplectic mass was exceedingly soft and flabby, much more like a clot of blood. It would scarcely bear to be incised, but broke down under the scalpel or finger as easily as, and indeed with a striking similarity to, the coagulum of blood which has stood for a time after venesection.

It would appear to me, that this softness of the diseased part, in this and similar cases, might be accounted for by the fact, of so great a quantity of blood having been effused into the pleural sac, which had thus escaped coagulation under that degree of compression, to which, in ordinary circumstances, it is exposed, being confined by the serous covering which encompasses the lung. This compression, existing as it does in the great majority of instances, may, in my mind, principally tend to produce that firmness generally presented by specimens of pulmonary apoplexy; but being to a great degree removed in the present case, by the laceration of the pleura, an opposite effect was produced.

Again, the entire superior lobe of the lung was engaged, so that I should be rather inclined to consider this as an instance of the uncircumscribed form of the disease, noticed by my friends, Drs. Graves and Wm. Stokes; for the line of separation between the superior and middle lobes, was the exact line of demarcation between the diseased and sound lung.

But what most attracted our attention, and, in my mind, gives its peculiar interest to this case, was, that in the superior and posterior part of the affected lobe, we found a laceration of the investing pleura, of about one inch in length, and half an inch in breadth, with very irregular edges, and immediately opening upon the point where the sanguineous effusion into the substance of the lung seemed most intense, and where we might naturally expect the greatest violence to be offered to its serous covering. This was evidently the source whence the blood, shed into the pleura, had come; for on examining as accurately as possible, we could discover the rupture of no vessel.

In this, and similar cases, are we to consider that the blood is the product of a general exhalation from the vessels of the part engaged? Are we to refer it to the rupture of a vessel of any considerable magnitude in the lung's substance? Or are we to attribute it in any, and in what, degree to that sudden expansion of the blood itself, to which Laennec refers, as being probably an influential cause?

This lung in other respects was healthy, though generally congested, as indeed was the right. The latter had contracted several old adhesions. The heart was sound and empty of blood. Blood of the body generally fluid." 15.

*Case* 2. Margaret O'Neil, aged 56 years, November 10th, 1828. She had complained, for two months past, of symptoms of chronic bronchitis, which were always relieved by purgative medicines. Yesterday she was seized with considerable hæmoptysis, which still continues. The pulse is 110, weak and small—countenance pale and anxious. The following indications were noted on examination.

"Percussion.—The entire chest sounds well, save the left subclavian region, which is dull.

Auscultation.—The respiratory murmur in the greater part of the superior lobe of left lung is either absent or very feeble; in points, a well marked rale crepitant is heard, and more particularly around the part where respiration is absent: puerile respiration in the rest of same lung, with heavy mucous rale about the leading bronchi. In the right, in spots the rale sonore is heard.

From this examination I could not hesitate in making my diagnosis, pulmonary apoplexy, and despite the age and considerable debility of the poor woman, I took sixteen ounces of blood from her arm, which induced faintness. I was encouraged in my decision to bleed her by observing, that the strength of the heart's action, as examined by the stethoscope, was disproportioned to the smallness and weakness of the pulse—an observation which, I well recollect to have first heard made, and in more instances than one practically illustrated by Laennec in his clinical wards of La Charité, and which I believe never should be omitted in deciding on a venesection in all cases of hæmorrhage. I prescribed a brisk purgative bolus and draught; and after their operation, a cough mixture, with three grains of tartar-emetic to the twelve ounces; two table spoonfuls to be taken every second hour. The strictest abstinence was enjoined." 17.

11th. Hæmoptysis greatly diminished—expresses herself relieved—bowels well opened—tolerates the emetic tartar. The stethoscope indicates no particular change since yesterday. Raised the dose of emetic tartar to six grains in the 12 ounce mixture. 12th. Hæmoptysis has almost ceased. The left subclavian region sounds clearer on percussion—and the râle crepitant is heard more feebly all over it. Pulse this day 96—she craves for food. From the 15th the convalescence of this patient was uninterrupted.

"The first of these two cases presents to our view a pathological fact of importance and great interest. It is one of, I believe, the very rarest occurrence. Laennec admits the possibility of such a thing taking place, but he never met it; nor am I aware of any such case being recorded in the annals of Medicine, save one by Corvisart, in his translation of Avenbrugger's work on Percussion.

In comparing the two cases together, we find a striking similarity in the general symptoms presented by each; except that in the former, the fatal case, the hæmoptysis was very slight; and it would seem to me that this very circumstance forcibly points out to us the value, nay, the necessity of a stethoscopic examination, to the formation of an accurate diagnosis. For it is undeniable, and the case of M'Cleary is a remarkable instance of it, that even a fatal degree of pulmonary apoplexy may be present without any, or with but very slight, hæmoptysis. Though the general symptoms, on which I am satisfied an unfounded reliance is too often placed, as well in other diseases of the chest as the present, by no means, in my opinion, indicated the presence of so formidable a disease; yet am I convinced, that had I made a careful stethoscopic examination of M'Cleary on the day he first presented himself to me at the dispensary, and not contented myself with general symptoms, I should have detected some traces of sanguineous engorgement of the lung, sufficient, unquestionably, to have induced me to adopt a line of treatment different from that pursued.

Granting the possibility that all the symptoms which this man presented on Friday, including the hæmoptysis, might be present without the existence of pulmonary apoplexy, and consequently that a stethoscopic examination on that day might have thrown no light on the case; yet would I ask, is it not much more probable, nay, does it not amount almost to a certainty, that the disease had been for some days making its insidious advances; that the day he was seen at the dispensary points of pulmonary apoplexy already existed, and that the excessive effusion of blood, which was so violent as to rupture the pleura, and induce fatal hæmorrhage into its cavity, was superadded to previously existing disease; the sudden effect of some unknown cause, perhaps a rush of blood to the superior parts of the body, to which the attitude in which he was seized, namely, stooped down to tie his shoes, was most favourable? And if we admit this, which, I must confess, the whole bearing of the case almost demonstrates to me, a stethoscopic examination made on Friday would have enabled me to form a diagnosis, which would, no doubt, have suggested the employment of curative means, calculated if not entirely to avert the attack of Sunday, at least to render it, I think it but fair to infer, amenable to active treatment." 20.

Dr. Ferguson appends some useful observations on venesection in this formidable disease. This is, no doubt, the most powerful remedy we possess; but, in few diseases is its employment less markedly indicated by the general symptoms—hence the greater necessity for, and utility of, auscultic investigation. For one case of this disease, where the symptoms of plethora are unequivocal, we meet with many where every symptom contra-indicates sanguineous depletion. It is hardly necessary to remark that, under such circumstances, the practitioner is thrown off his guard—pursues a wrong plan—and by tonics and stimulants, adds to the existing evil.

### III. *Case of Melanosis.* By John Crampton, M.D. &c.

The patient, D. Brown, a weaver, aged 34 years, was admitted into the Whitworth Chronic Hospital, on the 28th June, 1828, labouring under ascites to a great extent, the disorder being of six years' standing. A part of the abdominal distention evidently was owing to enlargement of the liver, the margin of which could be traced into the left hypochondre, which it completely filled, extending down, on each side, nearly to the spine of the ilium. Several tumours were felt on its surface—there was no pain; but some incumbrance from an enlarged organ. The breathing was laborious; but the lungs appeared sound on auscultatic examination. He had been very intemperate in the use of whiskey. Dr. C. considered the case as one of tuberculated liver, with consecutive ascites, and therefore fatal. On the 26th July, the distress from distention was so great, that paracentesis was performed, and four gallons of fluid drawn off, affording instant relief—but next day he complained of "soreness inside the belly"—and the tumours could now, of course, be more distinctly felt than ever. He died a few days afterwards, the pain and soreness gradually increasing—so that the immediate cause of death appeared to be peritonitis—no very infrequent consequence of tapping, in bad constitutions.

Dissection. "Thorax.—The pleuræ were studded with black tumours, each about the size of a pea; when cut into, they resembled coagulated blood; the lungs and bronchia were perfectly sound, and the heart natural.

Abdomen.—On opening this cavity upwards of a gallon of fluid was discharged. The surface of the contents of the abdomen was covered with immense flakes of lymph, which appeared quite recently formed. This adhered every where in bands, and glued the intestines together and to the liver. The peritoneum was of a brownish colour, covered with a false membrane, which was easily scraped off with the knife.

The mucous membrane of the stomach was very vascular, that of the intestines did not present any thing very remarkable, their serous covering, however, was much thickened.

Nearly two-thirds of the abdominal cavity were occupied by the liver; the right lobe extended up into the corresponding side of the thorax, compressing the lungs on that side to half their natural dimensions. This immense viscus stretched across and occupied the whole epigastrium, and reached fully two inches below the umbilicus to within an inch of the spine of the ilium on both sides. On its surface it was thickly studded with round black tubercles, varying in size from that of a small plumb to that of a middle sized apple. When cut, these tubercles presented a soft pulpy matter, easily broken down, and resembling Indian ink in colour, staining every thing which came in contact with it. The interior of the liver was also studded with these black tubercles, so that when cut, the surface resembled a slice of plumb pudding.

The liver weighed 19 pounds; its circumference was equal to 3 feet 8 inches. Transverse diameter of the under surface 15 inches; antero-posterior diameter along the horizontal fissure 10 inches; that of the right lobe 12¾ inches; that of the left lobe 11½ inches.

The spleen was healthy in structure, but smaller than natural, having been compressed by the liver.

The left kidney contained several calculi in separate cysts, some black, like coal, others of the uric acid kind. Two similar ones were seen in the right kidney. These calculous concretions were found in the tubular portions of the kidney." 26.

Melanosis is not a common disease; nor is it accompanied by any symptoms which enable us to discriminate between it and some other organic changes. Laennec considered it an "accidental tissue"—but Andral objects to this, affirming that it is completely inorganic, and may exist in various forms. In chemical analysis melanosis appears to differ little from the blood itself. We have no knowledge of the mode in which this disease is formed—nor of any means of arresting its course.

IV. *Case of Perforation of the Stomach, and of the escape of a Lumbricus into the cavity of the Abdomen.* By Dr. Crampton.

Jane Elders, aged 50, was received into the Hardwicke Hospital, on the 24th November, 1828, as a fever patient, having been ill for several days—her bowels constipated—vomiting—intense pain in the abdomen—inflation and tenderness—dry tongue—sharp pulse, &c. The application of leeches stopped the sickness—and aperients cleaned the tongue. On the fifth day after reception, the pain, tenderness on pressure, and frequency of pulse continued, with the addition of diarrhœa. The latter ceased in a few days, but the debility became excessive—she wasted rapidly, and death put an end to her sufferings on the 7th December. On cutting into the abdomen a quantity of puriform matter gushed out, which appeared to have been secreted by an inflamed peritoneum, which was covered by false membranes, and highly phlogosed. The inflammation had spread to the pericardium, into whose cavity pus was effused.

"Upon pressing the liver downwards to push out the matter contained in the abdomen, a large lumbricus worm was squeezed out from the space under the liver occupied by the stomach. Upon examination a round perforation was discovered about the middle of the lesser curvature of the stomach. Externally its margin was smooth and defined, and lined by a membrane which appeared to be a continuation of the mucous membrane of the stomach; internally its margin was also defined, and surrounded by a hard elevated edge, irregular, somewhat triangular, enclosing an aperture into which the worm can be inserted. On the top of this elevated margin one small round ulcer was observed, about the size of a pea. The pylorus was perfectly healthy, the folds of the mucous membrane of the stomach were much larger than usual; but the membrane, except round the perforation, retained its healthy colour and consistence.

The whole tract of the intestinal canal, both in its serous and mucous coats, was intensely inflamed. Its entire course was carefully examined, and although containing a quantity of fæces, no worm could be detected." 31.

It is probable that organic disease existed in the stomach, which led to the perforation. It is curious that none of the contents of the stomach had escaped into the abdominal cavity. Dr. C. accounts for it, by supposing that, in the act of vomiting, the perforation was pushed against the liver, which acted as a valve, and prevented the ejection of its contents into the general cavity.

V. *Case of an Anomalous state of the Heart.* By Dr. J. Crampton.

In a boy, aged 10 years, who died dropsical, accompanied by symptoms of cardiac disease, intimated by palpitation, a pale livid colour, and a very distressed state of respiration, the following anomalous condition of the heart was found.

"On opening the thoracic cavity, the heart was seen to preserve its natural situation, its shape however attracted notice; the upper part of the right ventricle being pushed out in such a manner, as to present externally somewhat of a sugar-loaf shape. On introducing a finger into the pulmonary artery, this vessel was found to be considerably contracted at its entrance into the heart, and appeared to be *destitute of valves*. A finger introduced into the aorta entered freely into both ventricles.

The left ventricle was opened by cutting from the orifice of one of the pulmonary veins, thus laying open auricle and ventricle together. It was then seen in the auricle, that the *foramen ovale was open*. The ventricle was perfectly natural, with the exception of a slight increase in the thickness of its walls; it communicated in the usual way with the aorta, the valves of which were perfectly natural, and it also communicated with the *right ventricle*, in consequence of a deficiency in the upper part of the septum.

On slitting down the right side of the heart, from the superior vena cava, nothing remarkable was observed in the auricle, except its communication with the other auricle through the *open foramen ovale*.

The right ventricle was dilated opposite the deficiency in the septum, into a pouch which corresponded with the sugar-loaf projection observed externally. The pulmonary artery was found to communicate with a separate cavity, bounded by distinct walls, which however was attached to the right ventricle, and communicated with it by means of an opening capable of admitting the little finger, formed under the columnæ carneæ of the ventricle. The lining membrane of this cavity, as well as of the pulmonary artery, was spotted with lymph in several places, which, in the artery, assumed the appearance of warty excrescences.

The pulmonary artery was perfectly destitute of valves; its lining membrane, at the usual situation of the valves appeared a little puckered, constituting the contraction felt on the introduction of the finger. Above the contraction the artery was considerably dilated, and it was in this dilated portion that the warty excrescences were situated.

The symptoms in this boy, James Caffry, who was admitted into the Whitworth Hospital, 25th August, 1828, were the following:—

Complexion livid and sallow; circumscribed flush on the cheeks; face, legs, and whole surface of the body extensively anasarcous; he had been subject to palpitations from his birth, with pain in the left side; breathing very laborious; could not lie on either side, but was easiest sitting up. Pulse at the wrist 140; small and synchronous with the heart's action, but subsequently both gave an irregular and intermitting beat. On inspecting the thorax externally, an obvious difference was observable, and on measurement the right side exceeded the left by half an inch.

On percussion the thorax sounded tolerably well throughout. With the stethoscope, the respiration was generally pure; inferiorly and anteriorly at the left side it was completely masqued by a very loud *bruit de soufflet*, which was also distinctly audible in every part of the thorax and in the neck, but not over the brachial or femoral arteries. The noise also masqued altogether the sound of the heart's action, but its impulse was felt with the instrument. At times, during the course of the treatment, the impulse was tremendous. The *bruit de soufflet* some days was more overpowering; on others it was less so, according to the state of quietness of the patient, or the reverse. On one day a slight degree of *bruit de lime* was heard.

He lived until the 21st September, the dropsical effusion was in part removed, and the heart's action was less tumultuous for a considerable time before his death. A few days previously to this event, the palpitation returned with increased force, whilst his strength declined, and he suffered from diarrhœa. From these symptoms he experienced partial and temporary relief after a profuse perspiration. At length he sunk, worn out and exhausted; he signified, however, that he did not suffer pain. It is quite unnecessary to recite the detail of the treatment in a case so hopeless, as it was merely palliative." 37.

The chief interest in this case arises from the consideration that life should have been maintained for ten years, with an organ so imperfect as the heart was found to be.

For various other papers in this volume we must refer to other articles, and some to other departments of this journal, where they will receive a very full analysis, with the exception of a prodigiously lengthy paper of Dr. Corrigan, on the sounds and motions of the heart. We were never converts to his doctrines, and they are even now beginning to be forgotten —or at all events, to be disbelieved.

